# Array data extractor (ADE): a LabVIEW program to extract and merge gene array data

**DOI:** 10.1186/1756-0500-6-496

**Published:** 2013-12-01

**Authors:** Stefan Kurtenbach, Sarah Kurtenbach, Georg Zoidl

**Affiliations:** 1 Faculty of Health, Department of Psychology, Molecular and Cellular Neuroscience, York University, LSB 323A, 4700 Keele Street, Toronto, ON M3J 1P3, Canada

**Keywords:** Array Data Extractor, ADE, Gene expression array, Microarray data analysis, Meta-analysis, Combining of multiple datasets, Statistics, Merge p-values

## Abstract

**Background:**

Large data sets from gene expression array studies are publicly available offering information highly valuable for research across many disciplines ranging from fundamental to clinical research. Highly advanced bioinformatics tools have been made available to researchers, but a demand for user-friendly software allowing researchers to quickly extract expression information for multiple genes from multiple studies persists.

**Findings:**

Here, we present a user-friendly LabVIEW program to automatically extract gene expression data for a list of genes from multiple normalized microarray datasets. Functionality was tested for 288 class A G protein-coupled receptors (GPCRs) and expression data from 12 studies comparing normal and diseased human hearts. Results confirmed known regulation of a beta 1 adrenergic receptor and further indicate novel research targets.

**Conclusions:**

Although existing software allows for complex data analyses, the LabVIEW based program presented here, “Array Data Extractor (ADE)”, provides users with a tool to retrieve meaningful information from multiple normalized gene expression datasets in a fast and easy way. Further, the graphical programming language used in LabVIEW allows applying changes to the program without the need of advanced programming knowledge.

## Background

High-throughput gene expression array technologies are commonly used in biomedical research and provide huge amounts of data. Today, there are close to one million preprocessed datasets publicly available repositories like the NCBI Gene Expression Omnibus [1], ArrayExpress [2] or the Stanford Microarray Database [3]. This provides researchers with the opportunity to detect novel treatment targets for various diseases [4], discover and refine signaling pathways, and to identify unknown interaction networks. Combining and comparing data from different studies is a rewarding approach, but comparing data across several studies is a challenging task. Various approaches have been published to normalize and refine data to detect meaningful expression changes in genes/networks and there are several software packages, e.g. the open source software Bioconductor [5], allowing for complex microarray analysis like pre-processing, quality assessment, differential expression, clustering and classification, and gene set enrichment analysis. Other examples of open source software are the TM4 Microarray Software Suite [6] and GenePattern [7]. Whilst other software packages allow very advanced data processing, performing a meta-analysis with data from multiple studies and platforms is still difficult for a “bench” scientist, and there is a lack of user-friendly software allowing researchers do so in a fast and easy way. A remarkable online tool, INMEX [8], has recently been published, providing user-friendly web-based platform for meta-analysis, but other available tools require substantial bioinformatics skills perform cross-platform meta-analysis [8,9]. Here, we present a LabVIEW program, Array Data Extractor (ADE), which allows users to extract expression information for a list of genes from multiple datasets, merge it into one output file, and perform basic statistics. Although e.g. INMEX can perform much more advanced meta-analysis, ADE allows working offline with large datasets, easy modification of the code (see below), and to prioritize and exclude array spots according to their specificity.

LabVIEW is a graphical programming language, where code is written by wiring together graphical modules. While LabVIEW contains the same concepts found in most traditional programming languages, such as different data types, loops, variables, and object-oriented programming, the visual representation allows for easy access and modification of the code, in contrast to programming languages where the code is written in text. It must be noted that the user needs a licensed copy of the basic LabVIEW software. The LabVIEW code can be compiled into an executable file if wished. LabVIEW was chosen as the programming platform, because (I) the graphical programming interface allows users without profound programming skills to edit the program, (II) many processing subroutines (statistics, data sorting, fitting) are built-in and can be applied to the program, (III) it is platform independent, and (IV) LabVIEW is an established software platform used for various research purposes in many laboratories. Program structures can be assigned to existing projects, which is why several LabVIEW programs for various purposes have been published [10-18].

## Implementation

### Data extraction

**Figure 1:**
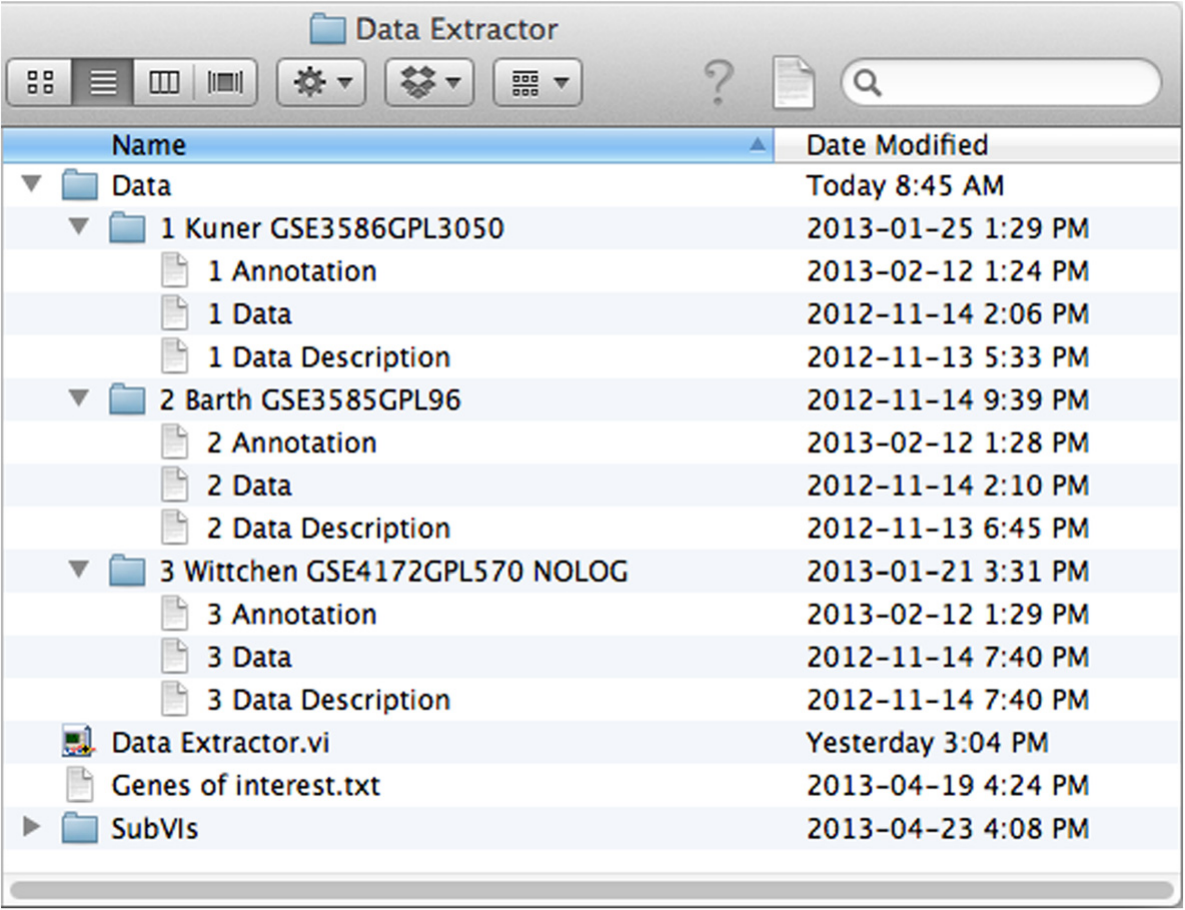
**File organization.** Files have to be organized as depicted. Folder names can be chosen differently, as long as they are numbered and numbers are separated with a space from rest of the name. If data in a study is non-logarithmic, user can include “NOLOG” in the respective data folder name. The folder SubVIs contains LabVIEW VI files that are required for ADE function.

The user provides basic information needed to process the data, organized as depicted in Figure 1. Sample files and detailed formatting informations are included in the supplementary files. First, a text file “Genes of interest.txt” has to be generated where the user defines the genes that he is interested in. Second, expression data has to be downloaded from e.g. the Gene Expression Omnibus (GEO) database. The user has to generate “Annotation.txt” files for each study containing information on how the genes of interest are named on the respective gene array (ID). GEO normally provides array annotations, or they can be obtained from the array manufacturer and copy-pasted into the Annotations.txt file. If wished, other unique identifiers like the Entrez Gene ID can be used instead, although in many cases the annotation files of the manufacturers are kept up-to-date and using gene names will make working with the output file easier. The user also defines the groups in the study (e.g. control, disease 1, disease 2) in the “Data Description.txt” file (see Additional file 1).

**Figure 2:**
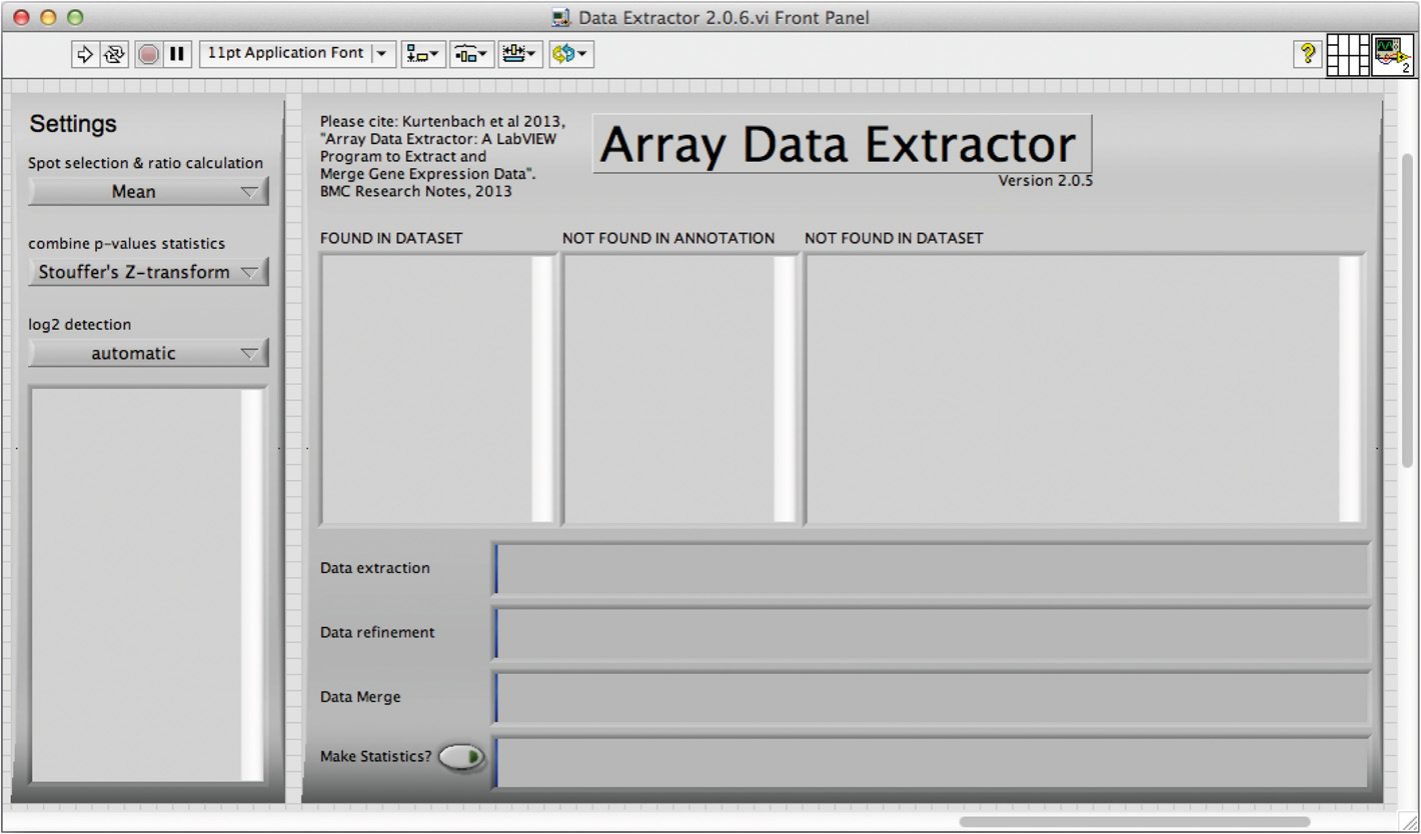
**Program interface.** While running, the program will indicate which genes were found in the dataset, which ones were not present in the array annotation (Annotation.txt file), and which genes are present in the chip annotation but not in the dataset. Progress bars indicate progress for data extraction, refinement, merging, and statistics. Statistics will only be performed when button is activated. In the settings tab on the left, the user can choose between median and mean values to be used for spot selection and ratio calculations. Further, Stouffer’s Z-transform method or Fisher’s method can be chosen to combine p-values from the studies. The user can also chose between automatic Log2 detection, or manual definition. In the latter case “NOLOG” has to be assigned to the folder names of the studies not Log2 transformed.

**Figure 3:**
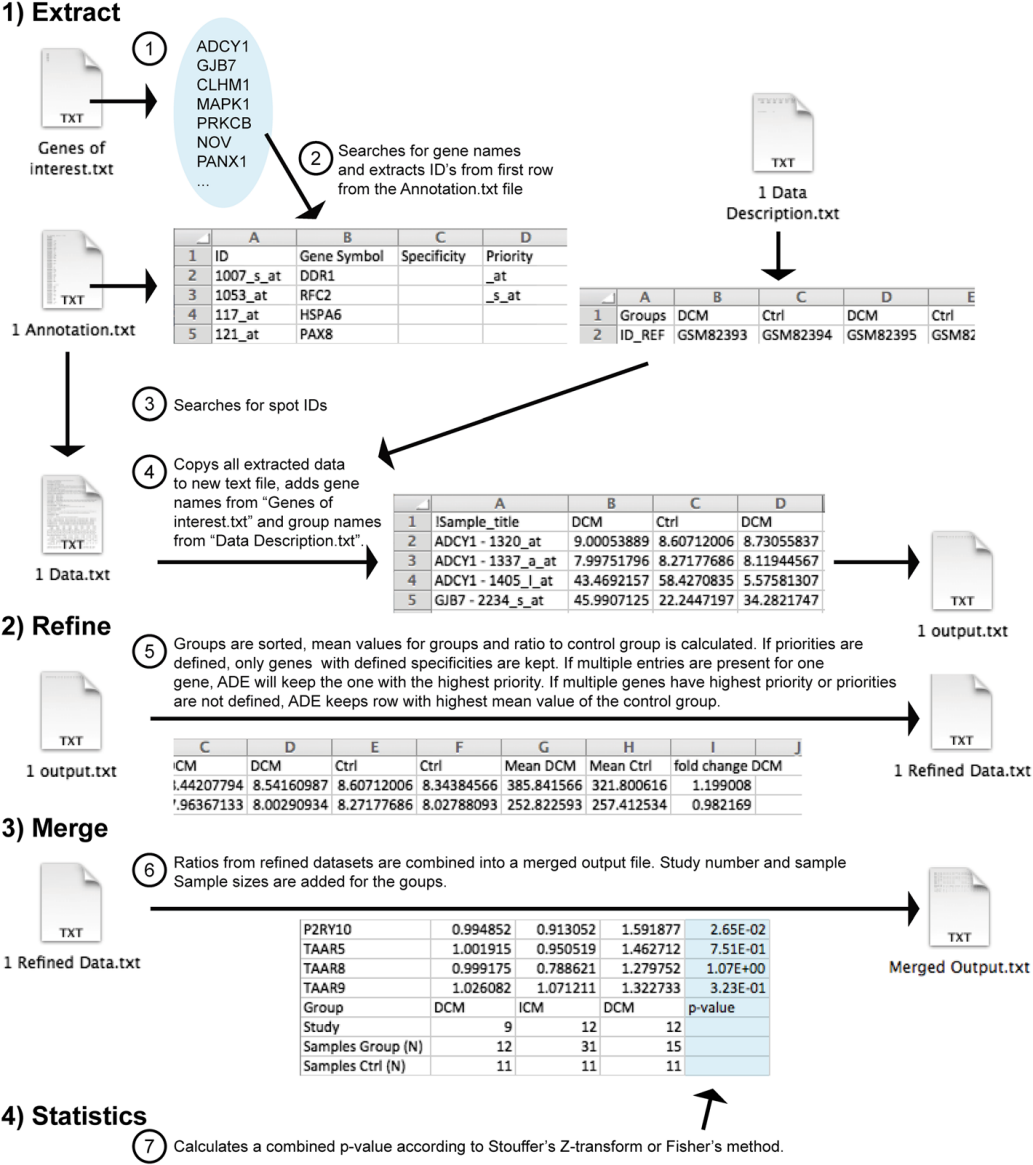
Overview over ADE workflow.

The program interface is depicted in Figure 2. Once started, ADE will perform a series of tasks, which are summarized in Figure 3. First, the software will extract all data for the genes defined (or e.g. Entrez IDs) in “Genes of interest.txt” from the “Data.txt” files. Extracted data will be saved in a new folder called “Extracted Data” for each study individually as “output.txt” files. Existing “output.txt” files will not be overwritten, as in some cases complete ADE runs are not needed, for instance when only one new study is added.

### Data refinement

Defined Groups will be clustered together correctly (because in some data files they are not) and a ratio of the means or medians (can be selected by the user on the interface) from the defined groups to the control group will be calculated. Because some studies provide their data non-logarithmic, the user can add “NOLOG” to the folder name of the respective study, which ADE will recognize, or turn on the automatic Log2 detection on the software interface. If automatic Log2 detection is turned on, ADE will display which studies it finds to be Log2 performed (please check if correct!).

In some gene expression arrays, multiple spots can be present for the same gene and there are several ways to deal with multiple probe sets [19]. ADE can automatically prioritize or exclude samples by their name on the gene array: Affymetrix platforms usually provide information about spot specificity in the spot ID name (e.g. “_s_at”). By adding a list of extensions to the fourth column of the “Annotation.txt” file (see Additional file 1) users can define which samples shall be included and in which priority. If information about specificity is available but not included in the probes names (e.g. for some Illumina arrays), the user can add this information in the third row of the “Annotation.txt” file. If there are multiple spots for one gene with highest priority (or if no priorities are defined), ADE will select the spot with the highest mean/median value of the control group by default. Refined data will be placed in a “Refined Data” folder. ADE will not overwrite existing files.

### Data merging

ADE will merge the ratio values for each gene and study into a single spreadsheet. Further, it will insert the correct group names, the study number, and information about the sample sizes (N). ADE will not overwrite an existing file, but perform statistics on it if selected (see below).

### Statistical analysis

If the “Make statistics?” button is activated, ADE will perform a statistical analysis of the data and provide a combined p-value for a gene being up- or down-regulated in all studies in the merged output file. As ADE will not overwrite existing files, it is possible to perform statistical analysis after an initial run, allowing the user to delete fractions (genes, studies, columns) or sort the merged output file before performing statistics (the first column, containing the gene names/IDs, and the column labels at the bottom of the file (for the remaining columns) must remain unchanged). ADE will first calculate p-values for each gene in the respective studies by performing a one-tailed Student’s t-test. To merge p-values, we included Stouffer’s Z-transform method [20] and Fischer’s method [21]. For Stouffer’s Z-transform method, p-values are first transformed to Z scores, with *Z*_*i*_ = Φ^- 1^(*p*_1_), Φ(⋅) being the standard normal cumulative distribution function (CDF). Z-Scores receive signs according to the gene being up- or down-regulated and summed to an overall Z-Score (Z_s_), with $Z_{s}=\sum_{i=1}^{N}Z_{i}/\sqrt{N}$, N being the number of Z-scores. Z-scores are not weighted in this approach. Z_s_ is finally transformed to a combined two-sided p-value (p_s_) with *p*_*s*_ = 2Φ (*Z*_*s*_). Fisher’s method uses $\chi_{2k}^{2}=-2\sum_{i=1}^{k}\mathrm{In}\left(p_{i}\right)$, where $\chi_{2k}^{2}$ is a chi-squared distribution with 2k degrees of freedom, k being the number of p-values. We found Stouffer’s method to be more restrictive, but other statistical approaches may also be considered [22-25].

## Findings

Hundreds of G protein-coupled receptors (GPCRs) are known to be expressed in the heart, some of which linked to heart disease formation [26-28]. To demonstrate ADE function, we downloaded a list containing 288 class A GPCRs from the International Union of Basic and Clinical Pharmacology database website [29] (http://www.iuphar-db.org) and expression data from 12 studies comparing normal and diseased human hearts (GSE3586 [30], GSE3585 [30], GSE4172 [31], GSE36961, GSE1869 [32], GSE32453, GSE29819 [33], GSE21610 [34], GSE9800, GSE5406 [35], GSE2656, GSE1145) from the GEO database. Datasets contained data for various disease groups. Downloading and organizing all files for ADE will take ~15 min per study initially, but once prepared files can be stored and reused for ADE runs. After preparation, ADE took ~5 minutes to extract and refine the data for all GPCRs on a standard desktop computer. Groups with less than 5 samples and genes covered by less then five studies were deleted manually from the merged output. Further, genes where <75% of the studies agreed on up-or down-regulation were excluded. P-values were calculated for the remaining 25 groups and 14 different heart disease conditions (for detailed information see Table 1). It may be considered to separate/exclude certain disease groups, but here we exemplarily performed statistics on the complete data. Statistical analysis took ~10 minutes to process.

**Table 1:** Class A GPCRs significantly regulated in human heart disease

**Gene**	**p-value**	**+/-**	**Gene**	**p-value**	**+/-**	**Gene**	**p-value**	**+/-**
P2RY13	0.00E + 00	+	NPBWR1	7.37E-08	-	GPR78	3.46E-05	-
P2RY14	0.00E + 00	+	PTGER2	7.82E-08	-	HCRTR2	5.29E-05	+
S1PR3	9.04E-52	-	P2RY12	7.98E-08	+	S1PR2	6.57E-05	-
GPR4	2.80E-31	-	GPR3	9.09E-08	-	OPN3	7.01E-05	-
P2RY2	3.08E-30	-	CMKLR1	1.07E-07	+	F2RL1	1.07E-04	-
ADRB1	7.41E-21	-	LPAR3	1.50E-07	-	HTR7	1.37E-04	-
MRGPRF	5.41E-19	-	P2RY11	1.68E-07	-	S1PR4	1.50E-04	-
C5AR1	3.34E-14	-	TAAR1	1.21E-06	-	GPR171	1.93E-04	+
PTGER3	3.91E-13	-	GPR84	1.25E-06	-	MRGPRX2	2.40E-04	-
GPR34	1.94E-12	+	GALR2	1.64E-06	-	GPR37L1	4.36E-04	-
CXCR4	1.77E-11	+	GPR161	2.88E-06	-	HRH2	4.47E-04	-
DARC	3.48E-10	-	RXFP3	3.60E-06	-	CCR2	7.51E-04	-
LTB4R	2.98E-09	-	TSHR	5.54E-06	-	BDKRB1	7.96E-04	-
FPR2	6.92E-09	-	ADRA1B	7.49E-06	-			
HTR2B	3.19E-08	+	P2RY6	8.14E-06	-			

47 of 288 class A GPCRs are differentially expressed in heart disease (p < 0.001). List of receptors was derived from the website of the International Union of Basic and Clinical Pharmacology (http://www.iuphar-db.org). Data was combined from 12 studies, containing 25 groups and 14 different cardiomyopathies: human arrhythmogenic right ventricular cardiomyopathy (ARVC, 1 group, 12 samples, 12 controls), dilated cardiomyopathy (DCM, 13 groups, 241 samples, 127 controls), doxorubicin induced cardiomyopathy (DOX, 1 group, 7 samples, 8 controls), fetal cardiomyopathy (FCM, 1 group, 5 samples, 5 controls), valvular cardiomyopathy (VCM, 1 group, 7 samples, 11 controls), hypertrophic cardiomyopathy (HCM, 3 groups, 119 samples, 55 controls), and ischemic cardiomyopathy (ICM, 5 groups, 166 samples, 52 controls). p-value represents combined p-values using Stouffer’s Z-transform method. +/- indicates up- or down-regulation reported by > 75% of the studies.

The analysis reported 43 class A GPCRs as differentially expressed in diseased human hearts with a p-value < 0.001 (Table 1), according to Stouffer’s Z-transform method. The confirmation of published experimental data demonstrates the functionality of the ADE software. For example, we found a highly significant down-regulation of the beta 1 adrenergic receptor (ADRB1) in 19/21 groups, which is well known for its crucial role in heart function and down-regulation in heart failure [36-38]. Other interesting candidates for in depth analysis were predicted, like P2Y receptor subunits, a receptor class expressed in various heart cells and regulating cardiovascular function in health and disease [39-46]. We also found a high significance for sphingosine-1-phospate receptor 3 (S1PR3) being down-regulated in 14/14 groups. Other S1PRs were differentially expressed with lower significances. S1PRs have multiple functions in the cardiovascular system including modulation of the heart rate, cardioprotection and vascular contraction [47-54]. A third interesting candidate among the ten most significantly regulated class A GPCRs is the prostaglandin E receptor 3 (PTGER3) in 21/22 groups. Activation of PTGER3 was shown to protect cardiomyocytes from oxidative stress [55] and reduce ischemia-induced arrhythmias and infarct size [56]. Overexpression was shown to promote hypertrophy [57] and changes in EP3 receptor density were reported after occlusion of the left anterior descending coronary artery [58]. These results exemplify how ADE can be utilized to quickly compare expression data as a starting point for further research. In summary, our analysis of diverse datasets from different heart disease groups strongly suggests that a substantial amount of class A GPCRs are significantly regulated.

## Conclusions

We introduce an easy-to-use software tool to extract and analyze normalized expression data. This program provides researchers with a tool to analyze gene array data utilizing publicly available normalized expression data. Beyond this scope, far more sophisticated tools (cited above) may be used for more detailed analysis.

### Troubleshooting

– Be sure to use “.” as decimal separators.

– Avoid duplicate gene names in “Genes of Interest. txt”.

– If ADE reports that it can’t open a file, be sure the format of files is correct (.txt), and/or use sample files provided to test.

– Moving Data folders between Mac and Windows systems may cause problems. Be sure to:

○ Delete the complete “Refined Data” and “Extracted Data” folders.

○ Check the Data Folder names, special characters may cause problems.

### Availability and requirements

**Project name:** Array Data Extractor (ADE)

**Project home page:** Software and sample data is included in the supplement. Webpage will be designed upon publication.

**Operating systems:** Mac OS X, Windows, and Linux

**Programming language:** LabVIEW (National Instruments)

**Other requirements:** LabVIEW (National Instruments)

**License:** GNU

## Competing interests

The authors declare that they have no competing interests.

## Authors’ contributions

Stefan Kurtenbach developed ADE. Sarah Kurtenbach validated program output and helped with the manuscript preparation. GZ helped with study design and manuscript preparation. All authors read and approved the final manuscript.

## Supplementary Material

Additional file 1 **Contains a text file describing how the files have to be formatted, aswell as the program and sample files.** Sample files include three data files downloaded from the GEO database [30,31]. With these files ADE can be directly tested without further modification. Click here for file
