# Supplementary material for: Array data extractor (ADE): a LabVIEW program to extract and merge gene array data
Source: BMC Res Notes. 2013 Dec 1;6:496. doi: 10.1186/1756-0500-6-496 (PMC4222097; doi:10.1186/1756-0500-6-496)
Supplement: Additional file 1 — Contains a text file describing how the files have to be formatted, aswell as the program and sample files. Sample files include three data files downloaded from the GEO database [30,31]. With these files ADE can be directly tested without further modification. [file 1756-0500-6-496-S1.zip › File Formatting.rtf]

File Formatting:All documents have to be arranged as shown in Figure 1. No additional files should be present in the folders. Data.txt files: Data.txt files can be downloaded and directly used e.g. from the GEO database. ADE will search in the first column for the ID, then copy the row for further processing. IDs cannot contain “_” else than for defining spot specificity. By default, ADE will ignore “ILMN_” in IDs. In the rare case there are other ID’s containing “_”, use a standard text editor to replace, or use the respective section in the LabVIEW code to automatically ignore terms. Annotation.txt files: The file has to contain four columns, labeled with “ID”, “Gene Symbol”, “Specificity”, and “Priority” (see example files in S2). IDs are the labels on the array, whereas “Gene Symbols” are the gene names, both are provided by the GEO database and the array manufacturer. If information on ID specificity is given in the ID names separated by a “_”, users can insert a list of priorities in the fourth column, starting with the highest priority desired. In this case, IDs not containing specificity indices indicated in the fourth column will not be included in the analysis. By default, ADE will only use spots with highest priority if multiple spots are present. If two or more spots have the highest priority, or no specificities are defined, ADE will only use the spot with the highest mean or median value by default. If IDs do not contain specificity information, users can add those to the third column. Specificity information has to be given either for all, or none IDs. ADE will add the specificity information to the ID name adding a ”_” between. Data Description.txt files: Files should contain two rows. Group names can be defined in row 1, whereas the names of the groups in the respective study have to be inserted in row 2. Group names have to have the same order as in the Data.txt file. Easiest is to copy the row from the Data.txt file and insert into row 2, then add group names in row 1. Note that one group has to be termed “Ctrl”. Additional rows do not impair ADE function and information about e.g. groups can be placed here if wished. Genes of interest.txt file: The file should contain a list of gene symbols in the first column. Avoid duplicate gene names. ADE will search for these genes in the Annotation.txt file, to retrieve the IDs in the Data.txt file. If wished, user can use Entrez IDs here instead (for both). For feedback, questions, bug reports or feature requests please feel free to contact me at any time at Stefan.Kurtenbach@me.com
